# Outcomes of Robot-Assisted Laparoscopic Prostatectomy with a Posterior Approach to the Seminal Vesicle in 300 Patients

**DOI:** 10.1155/2014/565737

**Published:** 2014-11-24

**Authors:** Takahiro Yasui, Keiichi Tozawa, Atsushi Okada, Satoshi Kurokawa, Hiroki Kubota, Kentaro Mizuno, Yukihiro Umemoto, Noriyasu Kawai, Shoichi Sasaki, Yutaro Hayashi, Kenjiro Kohri

**Affiliations:** ^1^Department of Nephro-urology, Nagoya City University Graduate School of Medical Sciences, 1 Kawasumi, Mizuho-cho, Mizuho-ku, Nagoya 467-8601, Japan; ^2^Department of Urology, Nagoya Tokushukai General Hospital, Kasugai, Aichi 487-0016, Japan; ^3^Department of Urology, Kainan Hospital, Yatomi, Aichi 498-0017, Japan

## Abstract

*Background*. The goal of this study was to analyze the perioperative outcomes of robot-assisted laparoscopic radical prostatectomies (RALPs) performed at our center. *Methodology*. We retrospectively reviewed 300 consecutive patients with clinically localized prostate cancer who underwent RALP with a posterior dissection approach to the seminal vesicle between May 2011 and November 2013. The mean patient age was 67.2 ± 5.5 years (range: 41–78 years), and the mean prostate-specific antigen (PSA) concentration, at diagnosis of prostate cancer, was 9.16 ± 6.50 ng/mL (range: 2.20–55.31 ng/mL). *Results*. The median duration of robotic surgery was 160 min (mean: 165 ± 40 min; range: 75–345 min). Median estimated blood loss, including that in urine, was 200 mL (mean: 277 ± 324 mL; range: 4–3250 mL). Intraoperative and immediate postoperative complications occurred in 3.0% of patients; 4 patients required allogeneic blood transfusion. As a measure of patient continence, 82.4% did not use more than 1 absorbent pad in 24 h, at 6 months postoperatively. *Conclusion*. RALP with an initial posterior dissection to the seminal vesicle was a safe and efficient method for controlling prostate cancer, even in these initial cases.

## 1. Introduction

Prostate cancer is the most common type of cancer affecting Japanese men, and radical prostatectomy is an established treatment option for both localized and locally advanced prostate cancers [1]. The development of minimally invasive surgical techniques has resulted in a greater focus on achieving optimal functional outcomes in patients undergoing this procedure. Laparoscopic radical prostatectomy (LRP) is an example of a minimally invasive technique for treating prostate cancer [2, 3] which is currently performed in Japan [4]. Compared to the open approach, surgeons with experience in LRP consider it advantageous because of the improvements associated with better optical magnification, less blood loss, less postoperative pain, and rapid resumption of normal activities [5, 6].

Despite the benefits of LRP, its use is declining worldwide. In the United States, it represents less than 5% of the total procedures used for treating prostate cancer, whereas robot-assisted laparoscopic radical prostatectomy (RALP) is now the most widely used procedure. In Japan, RALP has not been widely employed because of the lack of insurance coverage available for this technique, prior to April 2012.

Since the introduction of RALP in Frankfurt in 2000, there has been considerable interest in both its implementation and outcomes [7]. Robotic systems provide many advantages, including three-dimensional (3D) vision, enhanced magnification, tremor filtering, and motion scaling [8]. In addition, the EndoWrist technology aids in intracorporeal suturing and ergonomic comfort [8]. As any new surgical technique, RALP is associated with a learning curve in terms of operative outcomes (operating time, blood loss, hospital stay, and complications), oncological outcomes (positive margin rate and recurrence), and functional outcomes (incontinence and erectile dysfunction rates) [9, 10].

We aimed to evaluate the outcomes of the first 300 patients treated using RALP at our facility. We chose to focus on the total operative time, duration of robotic surgery, blood loss, intraoperative and immediate postoperative complications, duration of postoperative urethral catheterization, TNM staging, surgical margin status, urinary continence after surgery, and prostate-specific antigen (PSA) elevation recurrence. Furthermore, we compared the results of RALP with our previous LRP results.

## 2. Materials and Methods

Between May 2011 and November 2013, 300 consecutive patients were recruited for this study, each of whom underwent RALP at Nagoya City University Hospital. The study received approval from our institutional review board (“Robot-assisted laparoscopic radical prostatectomy for prostate cancer,” number 46-10-0009) and conforms to the provisions of the Declaration of Helsinki. Each patient provided informed consent. For all patients, the preoperative assessment included detailed patient histories, clinical examinations, serum PSA measurements, biopsy findings, Gleason score measurements, bone scan results, and contrast-enhanced computed tomography (CT) or magnetic resonance imaging (MRI) findings. Baseline demographic clinical staging was based on TNM staging (Union Internationale Contre le Cancer 2002 classification), and only patients with T_1–3_N_0_M_0_ stage cancers were considered for RALP.

All patients eligible for radical prostatectomy were offered RALP, using a 4-arm da Vinci-S robotic system (Intuitive Surgical, Sunnyvale, CA, USA). Our technique was based on that used at the Vattikuti Institute (Detroit, MI, USA), combined with the use of diathermy scissors [11]. Previously, we had performed LRP using a posterior approach to the seminal vesicle, according to the Montsouris method [2]. Therefore, the same approach was adopted for the RALP procedure [12]. Briefly, the rectum was retracted in a cephalad dissection by the assistant. The superior peritoneal arch (created by the impression of the Foley balloon) was grasped by the assistant or the third arm of the da Vinci-S and lifted upwards. A curvilinear incision was then created, using the monopolar scissors, midway between the anterior rectal wall and the grasped arch. Deeping of the incision by blunt dissection through the fibroalveolar tissue revealed both vas deferens (VDs). The VDs were dissected free, approximately 3 cm from the prostate, and transected. Blunt dissection of the anterior fibrovascular tissue overlying the seminal vesicles (SVs) continued laterally. Once the dissection was completed to the level of the base, blunt medial dissection freed the posterior surface of the SVs. After both SVs were completely dissected, upward traction on both SVs and VDs facilitated an incision into the Denonvillier's fascia, which allowed the posterior dissection to continue to the level of the rectourethralis fibers.

Dorsal vein control was achieved using 2-0 V-Loc on a 37 mm needle (Covidien, Mansfield, MA, USA) that was placed distally around the complex 3 times before division. Clipping of the vascular pedicles with Hem-o-lock (Teleflex, Limerick, PA, USA) 5 and 10 mm clips was used to control the posterolateral small vessels when performing (typically interfacial) nerve sparing. The use of the Rocco suture [13], using 3-0 V-Loc on a 26 mm needle (Covidien), was also adopted. Vesicourethral anastomosis was performed using two 3-0 V-Loc sutures on 17 mm needles (Covidien), tied together, forming a continuous suture running posteriorly and to either side. This also included an additional anterior racket stitch in cases with large bladder necks, according to the Vattikuti technique. After the first 70 cases, anastomosis was performed using two 3-0 PDSII (Ethicon Endo-Surgery, Cincinnati, OH, USA) sutures, retightened with Lapra-Ty (Ethicon Endo-Surgery) at the 3 and 9 o'clock positions. After the anastomosis was completed, a leak test was performed using 150 mL of saline, allowing an additional suture to be placed in the rare event of a leak. A 20 Fr 2-way silicon catheter was then inserted into the bladder. Cystography was performed between 5 and 8 days after the procedure, prior to catheter removal, unless the anastomosis failed the leak test.

Eight surgeons with experience in open radical prostatectomy and LRP conducted the procedures. The entire surgical team underwent 2 days of intensive training at Sukagawa Training Center in Fukushima or Fujita Health University Training Center in Aichi, Japan, and received surgical practice at the St. Augustine Hospital in Bordeaux, France, and/or the Yonsei University Severance Hospital in Seoul, Korea.

Side-specific intrafascial dissection of the neurovascular bundle was performed on prostates with palpable nodules, those with biopsy Gleason scores of 3 + 3 or 3 + 4, those with a maximum percentage of positive biopsy of <10% (depending on the location of the biopsy), and those with medially located positive cores or in the absence of suspected extracapsular extensions on MRI. However, prostates with a single positive biopsy and a Gleason score of 4 + 3 or those with a maximum percentage of positive biopsy of >10% comprising a medial core without signs of extracapsular extensions were also considered suitable for side-specific intrafascial dissection. These criteria were not considered strict rules but rather general guidelines [14]. Based on these specific indications, the number of patients who underwent nerve-sparing LRP (unilateral) was only 65. After the first 90 patients, we had been accustomed to robotic procedure, which showed stability after the RALP technique; all further intermediate and high D'Amico risk [15] patients underwent lymph node dissection.

Histopathological assessment included the final Gleason score, degree of positive margin, and SV or lymph node involvement. Pathological processing of the specimens included 4 mm sectioning of the whole gland; a positive margin was defined as the presence of malignant glands in direct contact with the inked surface. Patients were then followed up at regular intervals with serial PSA monitoring and assessment of functional outcomes, including continence and erectile function. For the 199 patients who were followed up for more than 6 months, we confirmed the continence rate by using a questionnaire at 1, 3, and 6 months after RALP.

For this study, the following data were collected and reviewed: patient age, body mass index (BMI), total operating time (including port placement, docking of the robot, dissection, anastomosis, and lymphadenectomy), duration of robotic surgery, estimated blood loss, hospital stay, presence or absence of urinary incontinence (pad usage), duration of postoperative bladder catheterization, intraoperative complications, immediate postoperative complications (appearing within the first month after surgery), long-term complications (appearing after the first postoperative month), TNM staging, and surgical margin status. Biochemical recurrence of prostate cancer, defined as increases in serum PSA levels of more than 0.1 ng/mL at 2 consecutive follow-up assessments, was also recorded. To examine the procedural learning curve, all variables were grouped for every 50 consecutive patients. Statistical significance was assessed using the Student's* t*-test and the Mann-Whitney* U* test.* P* values of <0.05 were considered significant.

## 3. Results

Preoperative data for all patients of RALP are shown in Table 1. Mean patient age was 67.2 ± 5.5 years (range: 41–78 years), and mean BMI was 23.3 ± 2.6 kg/m^2^ (range: 15.2–30.8 kg/m^2^). The mean PSA level at diagnosis of prostate cancer was 9.16 ± 6.50 ng/mL (range: 2.20–55.31 ng/mL). At biopsy, 96, 123, and 81 patients had a Gleason score of ≤6, 7, and 8–10, respectively. Further, 3, 73, 85, 32, 91, 12, and 4 patients had a preoperative clinical stage of T1a-b, T1c, T2a, T2b, T2c, T3a, and T3b, respectively. In 1 patient, open surgery was ultimately performed because of severe adhesions in the abdominal cavity after gastrectomy. One hundred forty-two of the 300 patients were initially diagnosed with localized prostate cancer elsewhere before being referred to our institution to undergo RALP; 16 of these patients had received neoadjuvant hormonal therapy at the first hospital. Eighty-seven patients underwent abdominal operations before RALP, the most frequent being appendectomy in 64 patients (21.3%), followed by cholecystectomy in 8 patients (2.7%) and gastrectomy in 4 patients (1.3%). The median follow-up duration was 15 months (range: 1–31 months).

The mean mass of removed prostate tissue was 44.4 ± 15.9 g (range: 14–132 g). The median total operating time was 220 min (mean: 222 ± 43 min, range: 120–410 min), with a median duration of robotic surgery of 160 min (mean: 165 ± 40 min, range: 75–345 min) (Table 2). Some patients required longer operative times because they had larger prostates, prostates that projected into the bladder, or adhesions to the surrounding tissue. The duration of robotic surgery remained stable despite increasing experience, after the initial 2 cases. There was no significant difference in the median duration of robotic surgery (155 min, 156 min, 178 min, 169 min, 172 min, and 162 min for cases 1–50, 51–100, 101–150, 151–200, 201–250, and 251–300, resp.). Each surgeon required some additional time during the initial cases.

The median estimated blood loss, including that in the urine, was 200 mL (mean: 277 ± 324 mL; range: 4–3250 mL). Eight patients showed blood loss of >1000 mL, but the hemoglobin levels, immediately after surgery, in 4 of these patients were >9 g/dL. However, 2 patients who experienced blood loss of 1250–3250 mL and 2 patients who developed postoperative hematomas required allogeneic blood transfusion. The volume of estimated blood loss tended to decrease with increasing surgeon experience, although there was major bleeding in some cases that did not otherwise exhibit any significant differences. There was no significant difference in the median blood loss; however, a tendency for slightly higher blood loss was observed in the initial 50 cases compared to the other groups (271 mL, 201 mL, 250 mL, 154 mL, 166 mL, and 150 mL for cases 1–50, 51–100, 101–150, 151–200, 201–250, and 251–300, resp.). The median duration of catheterization was 7 days (mean: 7.7 ± 4.2 days; range: 5–46 days), and patients undergoing RALP had a median postoperative hospitalization period of 11 days (mean: 10.4 ± 4.9; range: 7–50 days); the duration of hospitalization did not change with increasing surgical experience. One patient developed postoperative hematoma, infection, and ileus and required prolonged catheterization and hospitalization.

**Figure 1:**
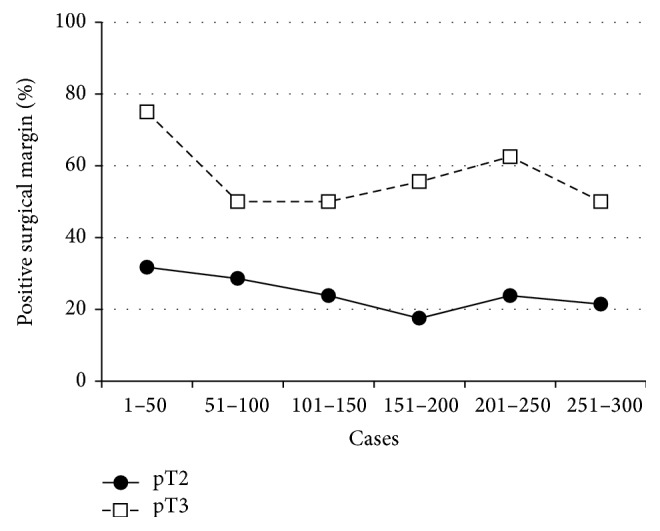
Percentage of positive surgical margins according to the pT category and surgical experience.

**Figure 2:**
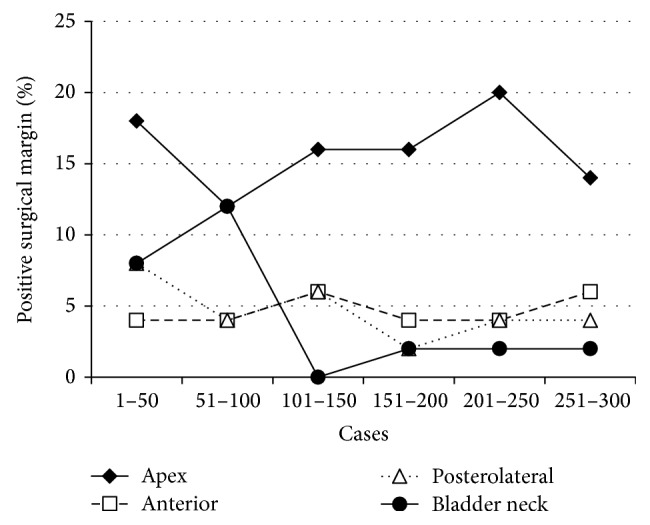
Percentage of positive surgical margins according to tumor location and surgical experience.

The number of patients in each pathological stage is given in Table 3. In 89 patients (29.7%), the surgical margins were positive. A change in the positive margin rate was seen over time for pT2 and pT3 cases (Figure 1). The rate of positive margins in pT2 cases was observed to decline with increasing surgeon experience; there were positive surgical margins in 31.7% of the first 50 cases and this declined to 21.4% for cases 251–300. There were no significant changes in the positive surgical margin rates in pT3 cases, but there were comparatively few such cases. The changes observed in the rates of positive margins, in both pT2 and pT3 cases, for different locations are shown as a function of the number of cases (Figure 2). Apical and posterolateral margins were generally seen most frequently. However, in cases 51–100, positive bladder neck margins were also frequently observed.

PSA recurrence was seen in 5 cases of pT3 cases and 2 cases of pT2 cases. Intraoperative and immediate postoperative complications occurred in 9 out of the 300 cases; the details are listed in Table 4. Three intraoperatively identified posterior bladder perforations were immediately sutured during laparoscopy. Four patients had postoperative hematomas requiring allogeneic blood transfusions.

Urinary continence was also assessed in 199 patients who were followed up for more than 6 months. To avoid subjectivity in assessment and to facilitate comparability, the number of absorbent pads used per 24 h period was documented. Of the 199 patients treated using RALP, 57.8% used a maximum of 1 pad per 24 h at 3 months postoperatively, and this percentage increased to 82.4% at 6 months. Sufficient erectile function for sexual intercourse, with or without augmentation using phosphodiesterase 5 inhibitors, was noted in 76.9% of the patients who underwent unilateral nerve sparing.

## 4. Discussion

The benefits of robot-assisted surgery are most apparent for areas of the body that are anatomically confined and difficult to access with open surgery, such as the deep areas of the pelvis. Because of this, robotic systems have been commonly used in urology, particularly for radical prostatectomy. Advantages include better ergonomics; scaled, filtered, and miniaturized movements facilitating more precise dissection and suturing; magnified, stable 3D vision; and a shorter learning curve than that for basic laparoscopy. Several studies have documented the positive short-term and long-term outcomes using this technology [16–18]. However, given the cost of the robotics, these systems are still relatively new in Japan and in developing nations with limited resources. We acquired the 4-armed da Vinci-S surgical system in 2011 and have been offering robot-assisted surgery to most patients with clinically localized prostate cancer since then.

The operative data for RALPs was compared to data for our first 160 LRP cases, which were performed between August 2000 and December 2006 (Table 2) [4]. Preoperative characteristics of patients who underwent LRP have no difference from those of RALP patients, except for biopsy Gleason score (Table 1). The operative times and blood loss with RALP were significantly lower than those observed with LRP. In addition, the need for blood transfusions and the frequency of severe complications (e.g., rectal injury) with RALP were also less than those observed with LRP. The duration of urethral catheter placement was similar between the 2 procedures; however, the length of hospitalization following RALP was less than that following LRP. There is no significance between postoperative pathological diagnosis of RALP and that of LRP (pT2: 83.0%, pT3: 16.3% in RALP, pT2: 78.1%, and pT3: 21.9% in LRP, resp.).

The mean patient age in the present study was older (67.2 ± 5.5 years) than that observed in prior studies conducted in Western countries. A study by Kaul et al. [16] reported a mean age of 57.4 years, and that reported by Tewari et al. [17] was 58.4 years. However, a similar average age of 63.2 years was reported by Patel et al. [18]. The older average age noted in the present study may be because of a lower overall incidence of prostate cancer among Japanese men resulting from racial and environmental differences. Currently, in Japan, PSA screening often triggers a diagnosis of prostate cancer, yet the mean serum PSA level of 9.18 ng/mL in this study was nearly 1.5 times that reported in many other Western studies; for example, the mean serum PSA value was 6.9 ng/mL in the series reported by Patel et al. [18]. The high PSA levels noted in this study may be attributable to the preponderance of stage T2 cancers, accounting for a steeper learning curve for T2 cancers than for T1c cancers. In the current study, MRIs were performed using a 3-Tesla system to detect prostate cancer. With this system, the performance of diffusion-weighted imaging is better than T2-weighted imaging for prostate cancer diagnoses [19]. We believe that the higher accuracy of the MRI system resulted in the accurate identification of the increased percentage of clinical T2 cases in our study. This was despite the fact that prostate size (44.4 ± 15.9 g) was similar to that reported in studies by Kaul et al. [16] (48.6 ± 12.1 g) and Tewari et al. [17] (45.3 ± 12.3 g). A consistent long-term oncological follow-up study should be conducted to better address this issue.

The median duration of the robot-assisted surgery was 160 min in this study. In a prior multi-institutional report, Schatloff et al. [20] reported a median operative time for RALP of 165 min among surgeons with a median experience of 460 cases. Our data show that a similar mean operative time was reached after the first 10 cases for each surgeon. A reason for this may be that surgeons involved in the present study had considerable prior experience in LRP (over 50 cases each). Others have reported shorter mean duration of robotic surgery of 122 min [16] and 130 min [18]. Patel et al. [18] also noted that the duration of robotic surgery decreased with increasing surgeon experience; it was 202 min for the first 50 cases and less than 100 min for the last 100 cases. In the present series, the operative duration did not decrease despite 300 cases of surgical experience using RALP. The reason for the apparent rapidity with which our surgeons reached this plateau in the length of the operation may be related to their prior LRP experience, the comparative ease with which RALP can be mastered as compared with LRP and the fact that only 8 surgeons perform the RALP operation.

The estimated median blood loss was also relatively high in the present study (276.5 ± 323.8 mL), with 4 patients (1.3%) requiring blood transfusions. The mean blood loss reported by Tewari et al. [17] was 160 mL and that reported by Kaul et al. [16] was 111 mL. A review of the outcomes reported by high-volume centers, including studies involving at least 250 cases, showed that the mean estimated blood loss for RALP was 164 mL [21]. In their first 100 cases, Mikhail et al. [22] reported a mean blood loss of 340 ± 238 mL. The reduced levels of blood loss are ones of the chief advantages of RALP over open surgery.

Only 1 case (0.3%), in which the patient had severe adhesions in the abdominal cavity, was converted to open surgery in the current patient series. Patel et al. [18] reported a conversion rate of 0.6% in their series of 500 patients, whereas Mikhail et al. [22] reported a 7% conversion rate in their first 100 patients. In our study, 2 cases with posterior bladder perforations were immediately sutured by laparoscopy and no rectal injury was encountered.

During RALP, many surgeons currently employ the modified Montsouris technique, as initially described by Menon et al. 2002 [9], with initial anterior prostate dissection [2]. We adopted RALP with an initial posterior dissection approach to the SV and VD [12]. Several advantages are offered by this initial dissection. First, the surgeon is offered a larger working area to dissect the VD and SV. The surgeon is, therefore, able to visualize the VD as it courses towards the internal inguinal ring and prior to its transection. The second benefit of an initial posterior dissection is the visualization offered by the absence of pooled blood. For surgeons who dissect the SV only after bladder neck transection, blood collects in the fossa created in the rectoprostatic space and hampers tissue visualization. Third, the most important benefit of the technique is the safe and reliable posterior bladder neck transection. By ensuring complete mobilization of the prostate, the surgeon can through the anterior layer of Denonvillier's fascia into the previously dissected space.

In a recent study by McNicholas [23], increased RALP experience resulted in a reduced occurrence of complications. In our series, both the overall complications and the major complications decreased significantly with increasing experience, reaching levels similar to those published in studies involving very experienced surgeons. The perioperative complication rate in the current study was comparable to that of most contemporary series [24–28], despite the fact that the surgeons involved in the present work had limited RALP experience. Each of the surgeons involved in this study had performed over 50 LRPs, and all underwent RALP training using videos, lectures, or hands-on experience.

Atug et al. [28] examined the positive surgical margins of the first 100 RALP procedures at their hospital, according to case numbers, and observed rates of 45%, 22%, and 11.7%, for the first, second, and third groups of patients, respectively. In another recent study, Menon et al. reported an overall positive margin rate of 25.1% in a series of 1384 patients. The current data revealed an overall positive surgical margin rate of 29.7%. One reason for this high value was the high ratio of T3 patients. In T2 and T3 patients, the positive margin rate gradually decreased with additional experience (Figure 2). The positive margin rate in our study was relatively high, especially for cases 51–100 and for tumors located at the bladder neck. In these cases, the prostate dissection was approached from the bladder neck, avoiding the large, inner bladder neck. During the cutting of the bladder and prostate, which may have moved to the side, the absence of tactile sense with the robotic system may have contributed to the high rate of positive margins. When the dissection approach was changed slightly, to the bladder side for cases 101–300, the rate of positive margins decreased.

Improvements in functional outcomes, such as continence and potency rates, because of the surgical experience have been reported in a number of prior studies. Despite varying outcome definitions in these studies, similar results have been found at 1-year follow-up examinations. Menon et al. [29] reported a 96% continence rate at a 6-month follow-up assessment. Similarly, Joseph et al. [30] and Krambeck et al. [31] reported 90–91.8% continence rates at a 12-month follow-up assessment. In our study, the first 100 patients had a slightly lower pad-free rate, but this rate gradually improved. The increase in continence rates observed with surgical experience was statistically significant, and we speculate that similar results can be achieved after 50 cases. Additionally, another reason for the low continence rate was that the age of the patients was higher. Similarly, several high-volume series have reported potency rates of 70–78% at 12 months after RALP [17, 18, 29, 32]. Although we observed relatively good outcomes, these outcomes are difficult to analyze because of both the small number of cases who underwent nerve-sparing LRP (total: 65 patients) as well as the preoperative low International Index of Erectile Function (IIEF-5) score (preoperative: 11.3 ± 8.1 points, range: 0–22).

In this study, 8 surgeons were responsible for the 300 cases. Although each surgeon had prior experience with more than 50 cases of LRP, each had limited prior experience with RALP. However, we conclude that the RALP procedure is easier to learn than LRP because, with only 300 cases, the surgeons were able to achieve levels of positive outcomes that are similar to those reported in the literature.

The da Vinci system used at our institution was the 15th such system installed in Japan; 120 Japanese hospitals have now adopted the use of this system. The rate of RALP operations had been low in Japan, as well as in other developing countries. Prior to April 2012, RALP procedures were not covered by Japanese health insurance companies and this is believed to have influenced the lack of widespread use of this procedure in Japan. With its proven advantages and the increasing skills of the surgeons, this technology is likely to gain further acceptance in the near future. However, a decrease in the cost of robotic surgical systems is essential for continued feasibility of this technique.

For any new treatment modality to gain widespread global acceptance, the outcomes need to be reproducible across various centers and patient populations. Although RALP is a validated treatment option for the management of patients with localized prostate cancer, all prior reports have come from high volume centers in Western countries. This procedure is already well established in both Europe and the United States. However, additional validation of results from hospitals outside these countries is necessary.

## 5. Conclusion

This study showed good perioperative outcomes for RALPs in the initial 300 cases performed at our facility. Surgical, oncologic, and functional outcomes all improved with increasing surgical experience, following a relatively short learning curve after transitioning from LRP. After the first 300 RALP cases, outcomes were similar to those reported at high-volume medical centers.

## Figures and Tables

**Table 1:** Pretreatment patient characteristics of robot-assisted laparoscopic prostatectomy (RALP), and laparoscopic radical prostatectomy (LRP) reported previously.

Variable	Mean ± SD (range)	
	RALP	LRP^#^
Patients (*n*)	300	160
Average follow-up (months)	14.2 ± 7.8 (1–31)	35.0 ± 8.2 (3–73)
Age (years) (range)	67.2 ± 5.5 (41–78)	67.3 ± 6.1 (48–82)
BMI (kg/m^2^) (range)	23.3 ± 2.6 (15.2–30.8)	23.0 ± 2.6 (17.6–29.8)
PSA (ng/mL) (range)	9.2 ± 6.5 (2.2–55.3)	10.6 ± 8.7 (4.1–34.9)
Biopsy Gleason score (*n*)		
≤6	96 (32.0%)	72 (45.0%)
7	123 (41.0%)	58 (36.3%)
8–10	81 (27.0%)	30 (18.8%)
Clinical stage (*n*)		
T1a-b	3 (1.0%)	0 (0.0%)
T1c	73 (24.3%)	79 (49.4%)
T2a	85 (28.3%)	39 (24.4%)
T2b	32 (10.7%)	42 (26.3%)
T2c	91 (30.3%)	0 (0.0%)
T3a	12 (4.0%)	0 (0.0%)
T3b	4 (1.3%)	0 (0.0%)
Previous abdominal surgery	87 (29.0%)	Unknown
Previous hernia surgery	14 (4.7%)	Unknown
Preoperative hormonal therapy	16 (5.3%)	Unknown

^#^Data from [4].

**Table 2:** Comparison of operative and postoperative data and complications between robot-assisted laparoscopic prostatectomy (RALP) and laparoscopic radical prostatectomy (LRP).

Characteristics	RALP	LRP^#^
Number of cases	300	160
Mean operative time (min)	165 ± 40^+∗^	296 ± 88
Blood loss (including that in urine) (mL)	276.5 ± 323.8^*^	541.3 ± 484.1
Transfusions (cases)	4 (1.3%)	7 (4.4%)
Conversion to open surgery (cases)	1 (0.3%)	5 (3.1%)
Mean time to urethral catheter removal (days)	7.7 ± 4.2	7.4 ± 4.3
Postoperative hospitalization (days)	10.4 ± 4.9^**^	14.8 ± 4.7
Complications		
Rectal injury	2	4
Ureteral injury	0	3
Bladder neck stricture	0	4
Subcutaneous hernia	1	2

^#^Data from [4]. ^+^Duration of robotic surgery. ^*^ *P* < 0.05 (unpaired *t*-test). ^**^ *P* < 0.05 (Mann-Whitney *U* test).

**Table 3:** Surgical factors and corresponding percentages of positive margins.

Variable	*n*	Positive margin cases (%)
Pathological Gleason score		
≤6	49	7 (14.3%)
7	206	66 (32.0%)
8–10	45	16 (35.6%)
Pathological stage		
pT0	2	0 (0.0%)
pT2a	54	5 (9.3%)
pT2b	9	2 (22.2%)
pT2c	186	54 (29.0%)
pT3a	26	15 (57.7%)
pT3b	23	13 (56.5%)

**Table 4:** Complications associated with extent of surgical experience.

Complications	Cases						Total
	1–50	51–100	101–150	151–200	201–250	251–300	
Intraoperative							
Rectal injury	0	0	0	0	2	0	2
Posterior bladder perforation	1	1	0	0	1	0	3
Immediate postoperative							
Hematoma	2	1^*^	1	0	1	0	4
Ileus	0	1^*^	0	0	0	0	1
Infection	0	1^*^	0	0	0	0	1

^*^One case had postoperative complications of hematoma, ileus, and infection.
